# Refinement of intraperitoneal injection of sodium pentobarbital for euthanasia in laboratory rats (*Rattus norvegicus*)

**DOI:** 10.1186/s12917-017-0982-y

**Published:** 2017-02-21

**Authors:** Katie K Zatroch, Cameron G. Knight, Julie N. Reimer, Daniel S. J. Pang

**Affiliations:** 1 Cornell University College of Veterinary Medicine, Ithaca, NY USA; 2 Veterinary Clinical and Diagnostic Sciences, Faculty of Veterinary Medicine, University of Calgary, 3280 Hospital Dr NW, T2N 4Z6 Alberta, Canada; 3 Départment de sciences cliniques, Faculté de medicine vétérinaire, Université de Montréal, Saint-Hyacinthe, Québec, Canada; 4 Groupe de recherche en pharmacologie animale du Québec (GREPAQ), Saint-Hyacinthe, Québec, Canada

**Keywords:** CCAC, Pentobarbital, Killing, Refinement, Welfare

## Abstract

**Background:**

The Canadian Council on Animal Care and American Veterinary Medical Association classify intraperitoneal (IP) pentobarbital as an acceptable euthanasia method in rats. However, national guidelines do not exist for a recommended dose or volume and IP euthanasia has been described as unreliable, with misinjections leading to variable success in ensuring a timely death. The aims of this study were to assess and improve efficacy and consistency of IP euthanasia.

In a randomized, blinded study, 51 adult female Sprague-Dawley rats (170–495 g) received one of four treatments: low-dose low-volume (LL) IP pentobarbital (*n* = 13, 200 mg/kg pentobarbital), low-dose high-volume (LH) IP pentobarbital (*n* = 14, 200 mg/kg diluted 1:3 with phosphate buffered saline), high-dose high-volume (HH, *n* = 14, 800 mg/kg pentobarbital), or saline. Times to loss of righting reflex (LORR) and cessation of heartbeat (CHB) were recorded. To identify misinjections, necropsy examinations were performed on all rats. Video recordings of LL and HH groups were analyzed for pain-associated behaviors. Between-group comparisons were performed with 1-way ANOVA and Games-Howell post hoc tests. Variability in CHB was assessed by calculating the coefficient of variation (CV).

**Results:**

The fastest euthanasia method (CHB) was HH (283.7 ± 38.0 s), compared with LL (485.8 ± 140.7 s, *p* = 0.002) and LH (347.7 ± 72.0 s, *p* = 0.039). Values for CV were: HH, 13.4%; LH, 20.7%; LL, 29.0%. LORR time was longest in LL (139.5 ± 29.6 s), compared with HH (111.6 ± 19.7 s, *p* = 0.046) and LH (104.2 ± 19.3 s, *p* = 0.01). Misinjections occurred in 17.0% (7/41) of euthanasia attempts. Pain-associated behavior incidence ranged from 36% (4/11, LL) to 46% (5/11, HH).

**Conclusions:**

These data illustrate refinement of the IP pentobarbital euthanasia technique. Both dose and volume contribute to speed of death, with a dose of 800 mg/kg (HH) being the most effective method. An increase in volume alone does not significantly reduce variability. The proportion of misinjections was similar to that of previous studies.

## Background

Over 2 million rats are used in biomedical research in Canada and the European Union annually [1, 2]. The overwhelming majority of laboratory studies employing rodents end with killing the animals upon completion of the study or if a humane endpoint has been reached. While this is a reality of research, efforts to refine killing methods, to achieve “euthanasia”, for rats and other laboratory animals are ongoing, as reflected in recent updates to the Canadian Council on Animal Care (CCAC) and American Veterinary Medical Association (AVMA) euthanasia guidelines [3, 4]. Goals for successful euthanasia include techniques requiring minimal restraint, simplicity of administration, and a swift, painless death [5, 6].

A commonly employed technique for euthanasia of laboratory rats is an overdose of carbon dioxide. However, current behavioral and physiologic evidence suggests that this method is aversive and may be painful [7–16]. As a result, the CCAC and AVMA have reclassified killing with carbon dioxide as “conditionally acceptable” [4] and “acceptable with conditions” [3].

In contrast, an acceptable method and preferred alternative to carbon dioxide is overdose with a barbiturate such as sodium pentobarbital (PB). An intraperitoneal (IP) route of injection is acceptable when intravenous injection cannot be performed or is impractical [3, 4]. Current guidelines do not indicate a specific dose of sodium pentobarbital for euthanasia, although 200 mg/kg or 3 times the anesthetic dose has been suggested [5]. There are several potential drawbacks associated with IP PB injection, including misinjection, variability in effect and pain [8, 17–23].

An important factor contributing to variability of drug effect (speed of onset and success) is misinjection, with deposition of injectate into intra-abdominal fat, abdominal viscera or the subcutaneous space. In the case of IP pentobarbital for euthanasia this results in a delayed time to death or even failure to cause loss of consciousness. Attempts to reduce variability with a two-person injection technique (one to restrain, one to inject) have had variable success, with reported proportions of misinjections ranging from 6 to 20% [19–21].

Pain, inferred from behavioral observations, necropsy findings and biomarkers, has also been cited as a potential impediment to achieving the principle of euthanasia. Specifically, exhibition of writhing (defined as the contraction of the abdomen and extension of the hind legs), grossly visible inflammation of abdominal viscera at necropsy and a measurable increase in spinal cord cFos have been reported following IP injection of pentobarbital [17, 18, 22, 23].

The primary aim of this study was to assess the impact of varying the dose and volume of sodium pentobarbital injected into the intraperitoneal cavity on time to death and consistency of the killing process. Secondary aims were identification of misinjections by necropsy and the quantification of writhing behavior in response to IP PB. We hypothesized that speed and consistency of IP euthanasia would be improved by using a higher dose and higher volume.

## Methods

### Study design

Fifty-one adult female Sprague-Dawley rats (170–495 g), sourced as surplus breeding stock, were included in the study. A sample size of approximately 13 animals, to achieve 80% power with an alpha of 0.05 (with an anticipated 20% misinjection rate) with an effect size of 1.5, was determined from pilot data. All animals remained in paired housing until the time of trial and were not handled prior to the study. Housing consisted of standard micro-filter cages (47 × 25 × 21 cm) with wood shavings and shredded paper bedding and a plastic tube for enrichment. A 12–12 h lights on-off cycle (lights on at 0700) was maintained in an environmentally controlled room (23 °C, 22% humidity). All experiments were performed during the light period (0730–1800).

Animals were randomly assigned to one of four treatment groups for IP injection. A low-dose low-volume group (LL, *n* = 13) received 200 mg/kg sodium pentobarbital (Euthanyl, 240 mg/ml, Bimeda-MTC Animal Health Inc., Cambridge, ON, Canada). A low-dose high-volume group (LH, *n* = 14) received 200 mg/kg sodium pentobarbital diluted 1:3 with phosphate-buffered saline (PBS). A high-dose high-volume group (HH, *n* = 14) received 800 mg/kg sodium pentobarbital. A control group (*n* = 10) received 1 ml of PBS. Each treatment was placed in a 1 ml (LL and control groups) or 3 ml (LH and HH groups) syringe as dictated by the volume of injectate. A new 25 G 5/8” hypodermic needle was attached to each syringe for injection. Blue food coloring (0.01 mL, Club House, Burlington, Ontario) was added to each treatment to facilitate visualization of injectate during necropsy examination.

At the beginning of each trial, a single rat was removed from the housing unit and placed in a Plexiglas chamber (L × W × H: 27.5 × 14.5 × 20.5 cm). Two video cameras (Panasonic HC-V720P/PC, Panasonic Canada Inc., Mississauga, ON, Canada) were placed along the long and short axes of the chamber. Before injection, baseline video of the rat was recorded for 10 min. Treatments were prepared in a separate room during baseline video recording. Individuals performing the IP injections and behavioral analyses were blinded to treatment.

**Fig. 1:**
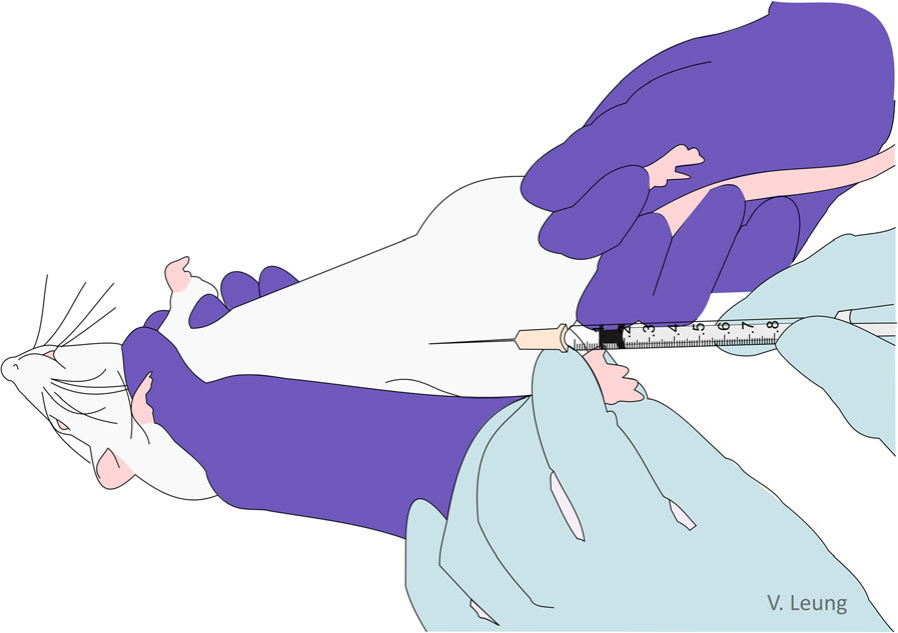
A cartoon showing the two-person injection technique used in the study, with one person holding the rat in dorsal recumbency (head down) and the second person gently restraining the right pelvic limb to facilitate intraperitoneal injection in to the right caudal quadrant

Following baseline video, each rat was removed from the box and restrained for a two-person injection technique. Rats were held in dorsal recumbency at an approximately 30° angle (head lowermost). The holder (DP) supported each rat and restrained the left pelvic limb. The individual administering each injection (KZ) restrained each rat’s right pelvic limb, injecting with the right (dominant) hand (Fig. 1). Each injection was performed in the right caudal quadrant of the abdomen at the level of the coxofemoral joint and approximately 5 mm to the right of midline. The needle was directed cranially at a 45° angle to the body wall.

Immediately following completion of injection, each rat was returned to the observation chamber. A single blinded observer (KZ) monitored for signs of ataxia (stumbling, falling, crossing feet) following injection. If signs of ataxia were noted, an attempt was made to place the rat in dorsal recumbency to evaluate for a loss of righting reflex (LORR), a surrogate for loss of consciousness [8, 24]. LORR was confirmed if the rat remained in dorsal recumbency for ten seconds. Failure of LORR was established if the rat resisted initial placement on its back or was able to right itself within ten seconds. In cases of initial LORR failure, the test was repeated every 30 s until LORR occurred. Following LORR, the animal was monitored for onset of apnea, defined as the animal’s chest ceasing to rise and fall. If and when apnea occurred, the rat was placed in left lateral recumbency. The left thoracic wall was then auscultated continuously with a stethoscope to identify cessation of heartbeat (CHB). Following CHB confirmation, video recording was stopped. The observation chamber was cleaned between trials.

When CHB did not occur within 20 min of IP injection, animals were euthanized with an overdose of carbon dioxide gas using a gradual fill (30% chamber volume per minute) technique. These cases were considered unsuccessful euthanasias.

### Necropsy examination

Following CHB, each animal was carefully removed from the chamber and positioned in dorsal recumbency for necropsy examination. The skin was incised along the midline and the injection site was identified in the abdominal wall musculature. The abdominal wall was incised and the intestines were reflected out of the abdominal cavity. Distribution of blue injectate and any misinjection into hollow viscera were noted. The liver was reflected cranially and any presence of dye within the biliary vessels caused by uptake of injectate from the peritoneal cavity and subsequent biliary excretion was noted. The GIT from the cardia to the descending colon was removed and any intestinal segments with dye-stained serosa were opened to confirm or rule out intraluminal misinjection. Misinjection was defined as the presence of blue injectate within hollow viscera or subcutaneous tissues, or staining the fur. For each rat, the serosal surfaces of the abdominal wall injection site, the caudate liver lobe, and transverse sections of at least three intestinal sections were examined histologically after formalin fixation for evidence of acute inflammation or swelling of mesothelial cells. Evaluation was performed by a single board-certified veterinary pathologist (CK), who was blinded to treatment group assignments.

### Off-line video analysis

Videos of the HH and LL trials were analyzed for the incidence of writhing behavior by a single individual blinded to treatment (JR). Baseline recordings were analyzed in their entirety while post-injection videos were analyzed until the rat became ataxic. Videos from the saline group were analyzed post hoc, with a viewing duration of 199 s; the mean time + 2SD for LORR in the LL group. As blinding was limited (two additional PB videos, one pre- and one post-injection, were added to introduce uncertainty), these videos were scored by two observers independently (JR, DP). Writhing was defined as a contraction of the lateral abdominal walls to the extent where the abdomen became concave with concurrent extension of the pelvic limbs [18, 23].

### Statistical methods

All statistical analyses were performed using commercial software (GraphPad Prism v.6.03, GraphPad Software, Inc. La Jolla, California, USA and IBM SPSS Statistics 21, IBM, Armonk, NY, USA). Data were considered approximately normal if skewness and kurtosis were less than ± 1.5 and 3, respectively. Between-group comparisons were performed with a one-way ANOVA with a Games-Howell multiple comparisons test. Consistency of the euthanasia process was assessed with a coefficient of variation (CV) calculation. Differences in median body weight between those animals in which a misinjection occurred and those with a successful injection were compared with a Mann-Whitney test. A *p*-value of < 0.05 was considered significant. Data are presented as mean ± SD.

## Results

Of 51 trials, 43 (84.3%) were successful IP injections and 8 (15.7%) were misinjections. Successful IP injections were distributed as follows: PB; *n* = 34 and control; *n* = 9. All successful injections with PB resulted in death: LL (*n* = 11), LH (*n* = 12), and HH (*n* = 11).

**Fig. 2:**
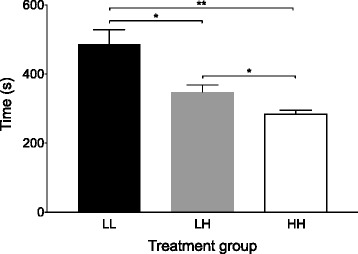
Time from delivery of the intraperitoneal injection to cessation of heart beat was fastest in the high-dose high-volume group (HH). LL = low-dose low-volume group, LH = low-dose high-volume group. *p < 0.05 **p = 0.002

The fastest killing method from injection to CHB was the HH group (283.7 ± 38.0 s), which was significantly faster than both the LL (485.8 ± 140.7 s, *p* = 0.002) and LH (347.7 ± 72.0 s, *p* = 0.039) groups (Fig. 2). Euthanasia in the LH group was also significantly faster than the LL group (*p* = 0.027).

The HH group was not only the fastest, but also the most consistent euthanasia method. The CV for HH was 13.4%, compared with 29.0% for LL and 20.7% for LH groups.

**Fig. 3:**
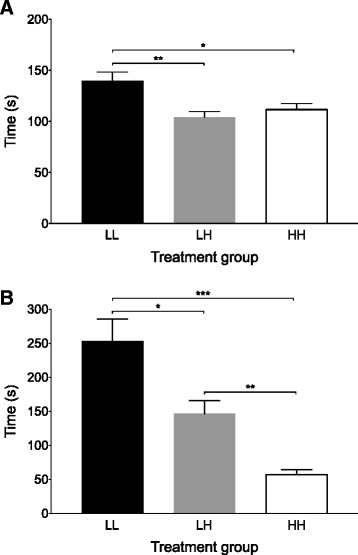
**a** Time from delivery of the intraperitoneal injection to loss of the righting reflex was longest in the low-dose low-volume group (LL). LH = low-dose high-volume group, HH = high-dose high-volume group. *p < 0.05 **p = 0.01. **b** Time from loss of the righting reflex to apnea was shortest in the high-dose high-volume group (HH). *p < 0.05 **p = 0.002 ***p < 0.001

The period from injection to LORR was longest in LL (139.5 ± 29.6 s), compared with both HH (111.6 ± 19.7 s, *p* = 0.046) and LH (104.2 ± 19.3 s, *p* = 0.01, Fig. 3a). Time from injection to LORR did not differ between LH and HH (*p* = 0.64). In no cases where LORR was confirmed were rats able to right themselves after 10s in dorsal recumbency had elapsed. The LORR-apnea time period showed the greatest variation between treatment groups and therefore had the greatest influence on the speed of the overall time to death (Fig. 3b). LORR-apnea was significantly faster in the HH group (56.8 ± 25.1 s) than LL (253.3 ± 106.7 s, *p* < 0.001) and LH (146.6 ± 66.1 s, *p* = 0.002). LORR-apnea in the LH group was also significantly faster than in the LL group (*p* = 0.03). There was no significant difference from apnea-CHB among treatment groups: HH (116.2 ± 19.7 s) versus LH (93.0 ± 29.0 s, *p* = 0.09), HH versus LL (92.9 ± 24.2 s, *p* = 0.06), LH versus LL (*p* = 1.0).

Eight misinjections were identified at necropsy. One misinjection was in a control animal. Seven misinjections were treatment group rats (HH; *n* = 3, LH; *n* = 2, LL; *n* = 2). Of these, euthanasia was unsuccessful (exceeding 20 min) in 3 (42.8%) animals (HH [*n* = 2], LL [*n* = 1]). In the four animals in which euthanasia was successful (HH [*n* = 1], LH [*n* = 2], LL [*n* = 1]), injection-CHB ranged from 318-1200s. There was no difference in body weight between animals with successful injections (325 [170–495]g) or misinjections (363 [285–461]g, *p* = 0.27, 95% confidence interval of the median difference; −24 to 90).

**Fig. 4:**
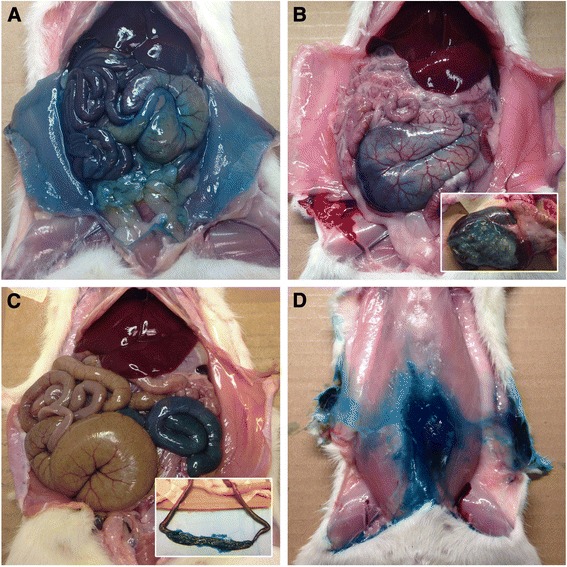
Abdominal cavities of four rats after confirmation of death; ventral view. Diffuse blue dye staining of serosal surfaces following successful intraperitoneal injection (**a**). Restricted dye distribution following inadvertent cecal (**b**), intestinal (**c**), and subcutaneous (**d**) misinjection. The insets in panels B and C show dye-stained ingesta, confirming inadvertent luminal misinjection

The anatomic distribution of the eight misinjections was as follows: four entered the cecal lumen, two entered the jejunal lumen, one was entirely within the subcutaneous tissues of the abdominal wall, and one was predominantly over the fur of the medial thigh, with a small amount in the subcutaneous space (Fig. 4). Cecal positions were variable: 14/51 (27.5%) in the right caudal quadrant, 5/51 (9.8%) located in the midline and 32/51 (62.7%) in the left caudal quadrant.

### Writhing

Writhing was not observed in the saline, LL or HH groups in baseline video recordings. Following injection, writhing, assessed in animals with successful injections, was seen in 45.5% (5/11) of HH and 36.4% (4/11) of LL rats. Two animals showed writhing behavior after saline injection (2/9, 22%).

## Discussion

Historically, concerns regarding IP euthanasia have revolved around misinjection leading to variable success rates and the potential for pain [8, 17–21, 23].

Our results show that: 1. IP injection with 800 mg/kg sodium pentobarbital (HH group) resulted in the fastest and most consistent killing method; 2. variable cecal position contributed to misinjections; and 3. the incidence of writhing behavior was less than half of that previously reported.

Both dose and volume contribute to the speed of euthanasia, and dose in particular appears to have the most dramatic effect on consistency of technique. The speed and consistency of the killing process can be improved through an increase in dose (accompanied by an increase in volume). Increasing injectate volume without increasing dose (LH group) improved the speed of IP euthanasia. However, further improvements in speed and consistency were achieved in the HH group.

From these results, several conclusions can be drawn. The mean + 2SD for the period from completion of injection to LORR was 151.0 s when 800 mg/kg pentobarbital (HH group) was administered IP. Therefore, it is highly likely that an animal that maintains LORR beyond this time has experienced a misinjection. If using the period from completion of injection to apnea as the indicator of successful injection, the time for mean + 2 SD was 259.1 s. Should these times be exceeded, a second injection of pentobarbital or alternative killing method should be performed.

Any increase in pentobarbital use is associated with an increased cost. For the formulation used here, this equates to approximately US$0.13 for the HH technique in a 250 g rat. While cost is an important consideration, it should be weighed against the labor cost of the slower (approximately 1.7 fold) LL group and potentially prolonged pain experience during the period from injection to LORR.

Misinjection is a consistent limitation of IP PB. The rate of misinjection in this study was consistent with the range reported in the literature (for injections given in to the caudal right abdominal quadrant), from 6 to 20% [19–21]. A factor contributing to the misinjection rate is variability in cecal position. IP injection is usually performed in the right caudal abdominal quadrant and previous work has confirmed that the cecum is most commonly located in the left caudal abdominal quadrant (61.9%, right 24.2%, middle 13.8%, total *n* = 289 adult male and female rats) [20]. Our results are similar to these findings despite using a different injection technique. In the study of Coria-Avila et al. [20], rats were restrained by a single person and suspended vertically by the thorax with the head up. This suggests that body position during injection has a minimal effect on the incidence of misinjection. Based on the misinjection rates in this and other studies, as well as the positional variation of abdominal viscera noted on necropsy, variations in IP injection methodology are unlikely to eliminate the possibility of misinjections. The wide range in body mass of the animals did not affect the incidence of misinjections, though statistical power to detect the small difference in body weights between treatment groups was low (approximately 20%). The majority (6/8) misinjections resulted from penetration of the cecal or jejunal lumen, suggesting that the presence of intra-abdominal fat did not hinder IP penetration.

Given this inherent obstacle in refining the euthanasia process, we hope that the recommendations described above will facilitate early identification of a misinjection, guiding the decision to repeat the injection or select an alternative euthanasia method.

We observed a substantially lower incidence of writhing behavior than previously reported and the reason for this discrepancy is unclear [17, 18]. Previous reports describe incidences of close to 100% whereas we observed writhing behavior in fewer than 50% of animals [17, 18, 23]. To facilitate comparison with these reports, we used the same definition of writhing as that described by Wadham (1996) and Ambrose (1998, 1999) [17, 18, 23].

The proposed cause of writhing behavior is the pain resulting from the alkaline pH of the PB solution. The pH of the solution studied here was 11.02 (measured independently by a commercial compounding pharmacy) within the range (pH 10.9–12.6) associated with a high incidence of writhing [17, 18, 23]. Current suggestions to alter the effect of pH focus on changing solution pH through buffering or the addition of lidocaine to provide analgesia [3, 4]. Wadham [23] reported that buffering a solution of sodium pentobarbital from an original pH of 12.6 to 9.4 resulted in precipitation [22].

Any study combining behavioral observation in the presence of drugs with sedative properties is inherently confounded by a reduced ability to express behaviors as sedation occurs. This is a limitation of the study design. The use of a vehicle control would address this, but one was not readily available as there were restrictions in obtaining formulation information from the manufacturer of PB. The dose we used in the LL group (200 mg/kg), was higher than that of Ambrose [17, 18] (150 mg/kg) and selected based on our institutional standard operating procedure. This may have contributed to the lower incidence of writhing observed by shortening the time after injection when writhing behavior could be expressed, before sedation occurred.

A lack of habituation to handling may have contributed to our findings. The rats used in this study received little or no handling prior to the experiment. Therefore, the stress associated with handling, injection and the observation chamber may have led to a suppression of normal behaviors. The choice to use Sprague-Dawley rats was based on local availability, through an institutional program to make available unused rats for research. As other strains were not studied it is impossible to account for strain differences in drug pharmacokinetics or pharmacodynamics.

Euthanasia can be performed or facilitated by inducing general anesthesia before a physical euthanasia method with drug combinations other than PB, such as xylazine-ketamine or ethanol (in mice) [25, 26]. The general principles discussed here, of ensuring that LORR and death occur within an acceptable time following injection, should be applied. It is likely that guidelines for identifying misinjection would need to be determined for each drug or drug combination, accounting for differences in speed of onset of action and differences in effect. Data available for intraperitoneal injection of 70% or 100% ethanol in mice suggests 45–60s (75th percentile) to achieve LORR and 3.0–4.25 min for apnea [25]. In contrast, the time to LORR is considerably longer with xylazine-ketamine, taking 6.8 min for LORR and 8.2 min for loss of the pedal withdrawal reflex (mean + 2SD for both) [26].

## Conclusions

By coupling the effects of volume and dose with the incidence of misinjections we have suggested practical guidelines to refine overdose with IP sodium pentobarbital as a killing method in rats. Of the groups studied, IP pentobarbital at a dose of 800 mg/kg (HH group) was the most effective, resulting in the fastest time to death with least variability.

## Abbreviations

AVMA: American Veterinary Medical Association
CCAC: Canadian Council on Animal Care
CHB: Cessation of heartbeat
CV: Coefficient of variation
HH: High-dose high-volume
IP: Intraperitoneal
LH: Low-dose high-volume
LL: Low-dose low-volume
LORR: Loss of righting reflex
PB: Sodium pentobarbital
